# Hereditary Autonomic Neuropathy of the Oral Cavity and its Management

**DOI:** 10.22037/ijcn.v15i4.32016

**Published:** 2022-01-01

**Authors:** Niloofar ESMAEILZADEH, Mahmoud Reza ASHRAFI, Hossein SHOJAALDINI ARDAKANI, Bahman SERAJ, Parissa AREF

**Affiliations:** 1 Department of Pediatric Dentistry, Faculty of Dentistry, Tehran Medical Sciences, Islamic Azad University, Tehran, Iran.; 2 Pediatric Neurology Department, Tehran University of Medical Sciences, Tehran, Iran; 3 Pediatric Neurology Department, Alborz University of Medical Sciences, Karaj, Iran; 4 Dental Research Center, Dentistry Research Institude, Department of Tehran University of Medical Sciences,Tehran,Iran

**Keywords:** Hereditary sensory and autonomic neuropathy, Oral ulcer, Self-mutilation.

## Abstract

Hereditary sensory and autonomic neuropathies (HSAN) are rare genetic disorders that often manifest during childhood in the form of absence of pain sensation or self-mutilation. Patients often present significant oral self-mutilation manifestations, and biting of the lips, tongue, and cheeks have been frequently reported. This case report describes a case of hereditary sensory and autonomic neuropathy with oral and cutaneous ulcers.

Our patient was a 14-month-old girl with the chief complaint of a tongue ulcer, as stated by her parents, who were referred to our private dental clinic. Clinical examination revealed severe ulcers due to biting (Riga-Fede disease) on the ventral surface of the tongue and superficial ulcers on the dorsal surface of the tongue caused by the anterior maxillary teeth, along with some sores on fingers. The parents were healthy, with no congenital disease or familial history of a similar condition. The electrodiagnostic test revealed the absence of sensory nerve action potential response. However, the electromyographic findings and the compound muscle action potential of the tibial and ulnar nerves were normal.

Oral ulcers such as trauma to the lips and tongue, and self-mutilation trauma to the fingers can be used for early detection of Hereditary sensory and autonomic neuropathies. A multidisciplinary approach involving a professional dental team and a regular treatment protocol are imperative to prevent complications of Hereditary sensory and autonomic neuropathies.

## Introduction

Hereditary sensory and autonomic neuropathies (HSANs) are a group of rare genetic disorders caused by different mutations that can affect the function, development, and survival of sensory and autonomic neurons. Previously, the diagnosis and classification of HSANs were based on the age of onset, the pattern of inheritance, and clinical manifestations. However, at present, the diagnosis is based on the detection of pathogenic mutations by the whole-exome sequencing technique. While the phenotypic presentation of the disease may be variable, the gene penetrance is 100%. The impaired sensation of pain and heat are among the prominent symptoms (1). Mental retardation, ulcers in the lips, mouth, arms, hands, and feet, osteomyelitis, and chronic bone infections are among the other consequences of HSANs (2,3). This group of neuropathies exclusively involve the sensory nervous system and are genetically and clinically variable (4-6). While there is no definite treatment for the disease, currently available options are mainly supportive and palliative (1). Dyck and colleagues were the first to classify HSANs based on the age of onset, the pattern of inheritance, and clinical and electrophysiological findings into five major categories (7). Type I is more common in the second and fourth decades of life and manifests as an autosomal dominant pattern of inheritance. An impaired sensation of pain and heat in the feet, ulcers in the soles, Charcot joints, burning sensation complaints, and variable levels of sensory neural hearing loss are among its symptoms. Type II has an autosomal dominant pattern of inheritance and commonly occurs during infancy or early childhood. The typical symptom is the absence of sensation of all sensory stimuli. Other characteristics include forgotten fractures, lytic lesions in the distal phalanx, acropathy, and variable levels of autonomic involvement. Type III or familial dis-autonomy is a rare disorder with an autosomal recessive pattern of inheritance characterized by autonomic symptoms like thermal and blood pressure alterations, pulmonary infection, excessive sweating, impaired sense of taste, alacrima, gastrointestinal problems such as dysphagia, constipation, and recurrent diarrhea, and absence of corneal reflex. Those suffering from type IV are pain insensitive and have no mental retardation and anhidrosis since infancy. While those with type V are indifferent to pain since birth, they have no other autonomic symptoms (3). The identification of types VI and VII is based on the Haga classification. Psychomotor development does not occur in type VI patients, and they have autonomic disorders, negative deep tendon reflex (DTR), respiratory distress syndrome, malnutrition, hypotonia, alacrima, and absence of pain and heat sensation. Self-mutilation, painless fractures, delayed motor development, gastrointestinal disorders, and pain insensitivity are the main characteristics of those with type VII (8). Type VIII has recently been introduced by Chenetal and is characterized by self-mutilation behaviors along with orofacial injuries, painless fractures, skin and bone infections, corneal injury, decreased pain and heat sensation, and absence of mental disability (9) (Table 1). Self-mutilation behavior in these patients is primarily related to the orofacial region (2). Its oral manifestations include tongue biting, lip biting, and cheek biting (3). 

Herein, we report a patient with HSAN, and the methods administered to prevent oral self-mutilation are also explained. 

**Table 1:** HSANs classification based on their characteristics (1,3,8,9)

Type I	Absence of pain and heat sensation, and fine touch in the feet, sole ulcers, complaining of burning sensation, Charcot joint, variable degrees of sensory neural hearing loss, periods of extremity pain (1,3) common between 2^nd^ to 4^th^ decades of life (3)
Type II	Severe absence of sensation of all stimuli, forgotten fractures, lytic lesions in the distal phalanx, and acropathy (3) Common during infancy and early childhood (3)
Type III	Autonomic symptoms such as thermal and blood pressure alterations, pulmonary infection, excessive sweating, impaired sense of taste, alacrima, gastrointestinal problems, absence of corneal reflex (3), and no impairment of fine touch (1) It commonly manifests during infancy (3).
Type IV	Pain insensitivity, mental disability, anhidrosis (3), and periodic fever (1). It is prevalent during infancy (3). The sense of fine touch and heat sensation are intact (1).
Type V	Indifference to pain since birth and absence of other autonomic symptoms (3), frequent fractures, tooth loss due to periodontal disease, and neuropathic joints (1)
Type VI	All patients are under three years of age, absence of psychomotor development, autonomic disorders, negative DTR, respiratory distress syndrome, malnutrition, hypotonia, alacrima, and absence of pain and heat sensation (1,8)
Type VII	Hyperhidrosis, self-mutilation, painless fractures, delayed motor development, gastrointestinal disorders, absence of pain and heat sensation, neuropathic joints, delayed wound healing (1,8)
Type VIII	Self-mutilation behaviors and orofacial injuries, painless fractures, skin and bone infections, corneal injury, decreased pain and heat sensation, and absence of mental disability (9)

## Case presentation

**Figure 1:**
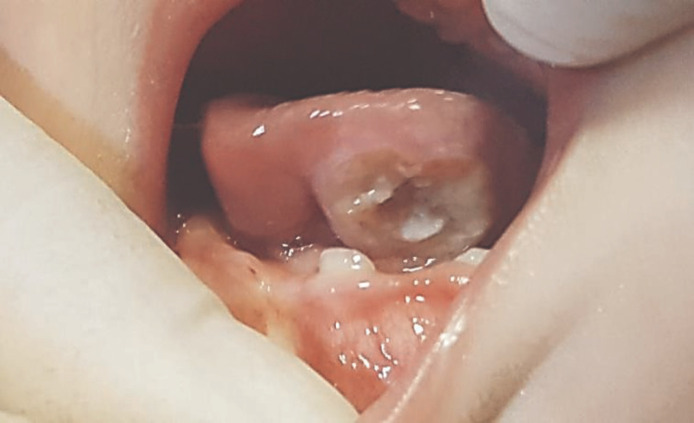
Tongue ulcer (Riga-Fede disease)

**Figure 2:**
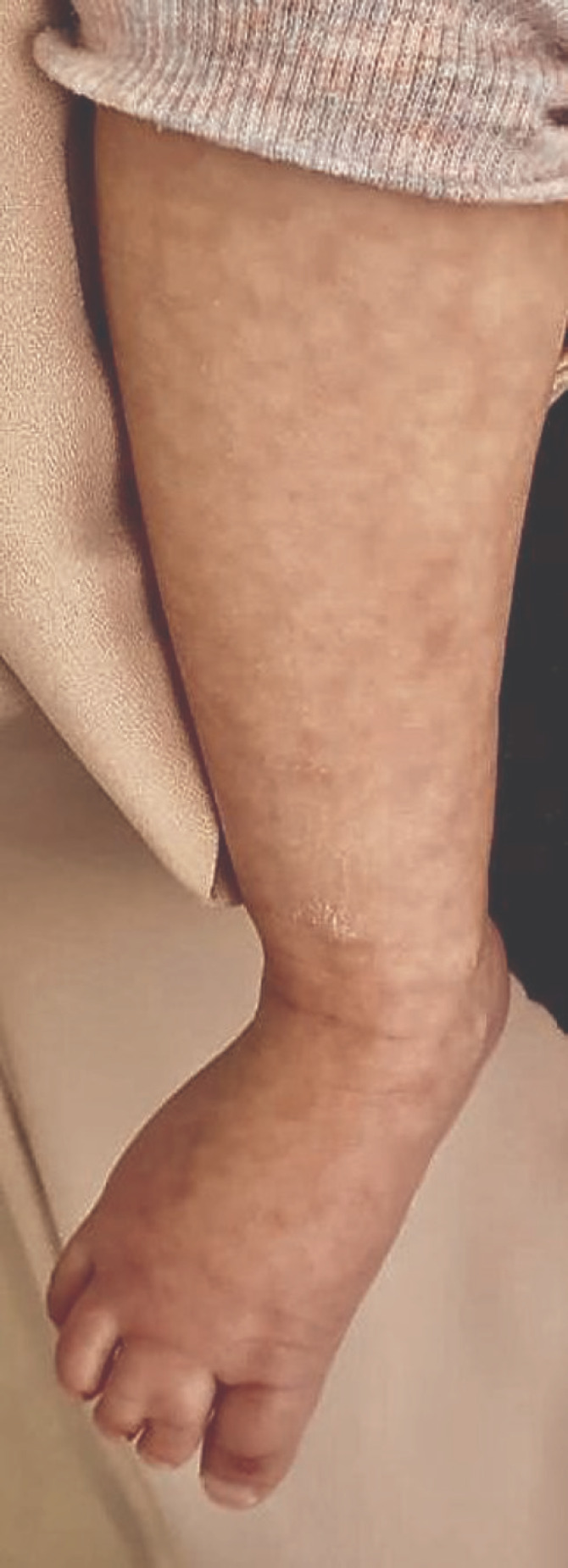
Mottling of the foot

Parents of a 14-month-old girl presented to our private dental clinic in June 2018 complaining of a large ulcer on the ventral surface of the tongue of their child (Figure 1). According to them, the child had started unintentional biting of her tongue since the eruption of her primary anterior teeth. She was the only child, and the parents had no consanguinity. The patient's medical history revealed mottling when cold, crying, or scared (Figure 2), delayed development, and general fatigue since birth. She had a history of aspiration of meconium at birth, lower than average height and weight percentile for her age, lack of growth of head circumference, excessive sweating from the neck up, history of corneal ulcers, severely impaired pain sensation in the extremities, gastrointestinal problems such as impaired food digestion and absorption, and decreased deep tendon reflex (DTR). According to the parents, she started holding her head up at the age of 5 months. She was followed up in one year. However, she did not start walking or speaking until the age of 2 years. She started crawling at the age of 2 years and six months and then started to say some words such as mom, dad, and water. At this time, she was able to crawl. The magnetic resonance imaging (MRI) revealed some degrees of advanced white matter disease, T2 signal changes in the deep and central white matter and corpus callosum, and progressive myelination in both the splenium and the genu, demonstrating T2 shortening. The DNA of the baby was then analyzed to assess the mutation of ten genes related to HSANs (i.e., WNK1, FAM134B, KIF1A, NTRK1, NGF, DNMT1, SPTLC1, SPTLC2, DST, ATL1) using the next-generation sequence (NGS) technique. The complete nucleotide sequence of these genes showed the absence of any mutation in the genes. Thus, the possibility of gene mutation as the etiology of this condition was low. However, it could not be definitely ruled out because mutations might have occurred in areas not detectable by the NGS technique. Alternatively, the mutation responsible for this phenotype might have occurred in some other genes that were not evaluated. Also, this technique does not include methylation changes, deletions/additions, and large chromosomal rearrangements. 

In the electrodiagnostic test, the compound muscle action potential (CMAP) was normal. However, the sensory nerve action potential (SNAP) was absent, and severe sensory neuropathies were reported in all extremities. Further examinations revealed that the patient was sensitive to thermal alterations; however, she was less sensitive to pain stimuli. 

**Figure 3:**
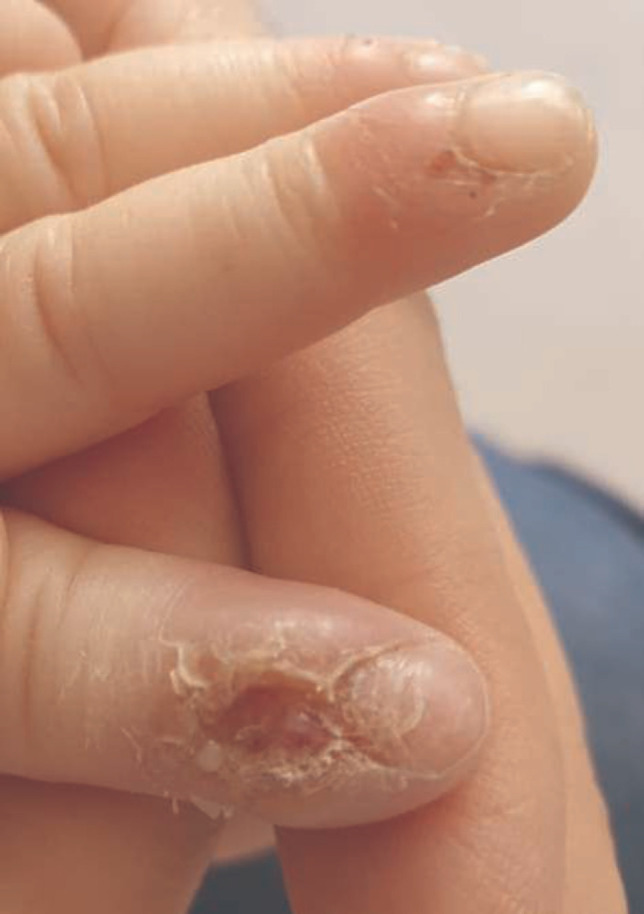
Ulceration of fingers due to biting

**Figure 4:**
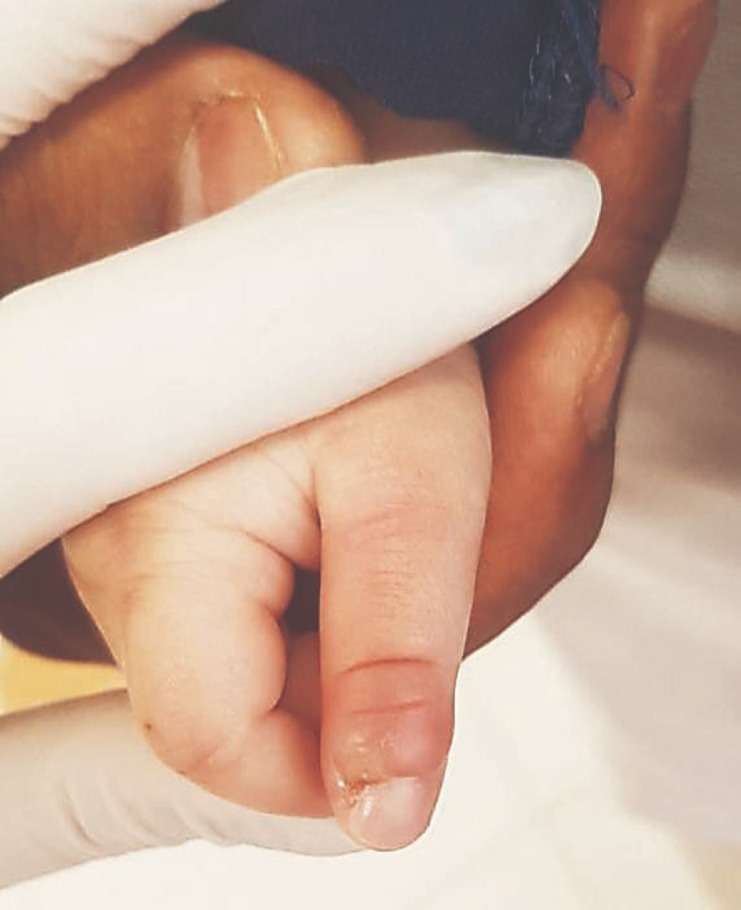
Ulceration of the thumb due to biting

**Figure 5:**
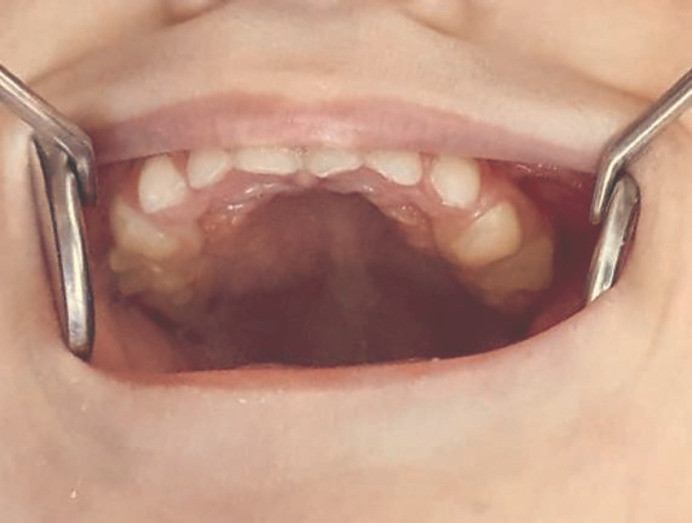
Grinding of the sharp edges of the maxillary teeth

**Figure 6:**
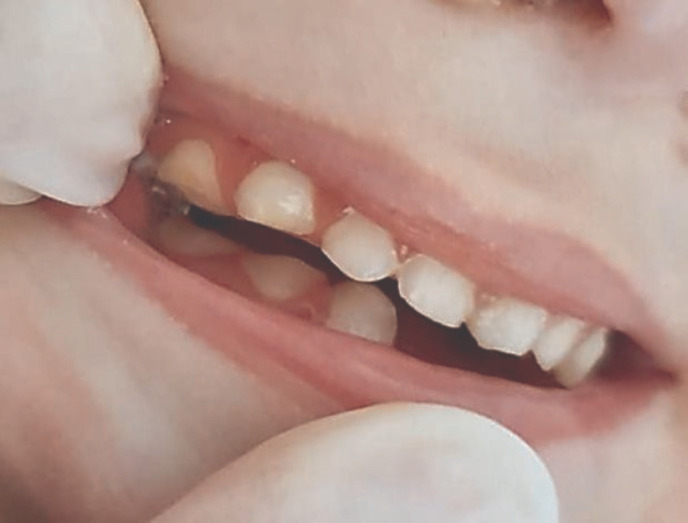
Posterior teeth of both jaws after grinding

Extraoral clinical examinations revealed self-injuries to the fingers that were healed after bandaging (Figures 3 and 4). Intraoral examination revealed severe ulcers (Riga-Fede disease) on the ventral surface of the tongue. To prevent the teeth from traumatizing the ventral surface of the tongue and avoiding trauma during bottle-feeding, the opening of the baby bottle was slightly dilated, which resulted in the healing of the tongue ulcers temporarily. However, in the next follow-up session, the mandibular anterior incisors and the left first molar had to be extracted due to severe trauma to the tongue. To preserve esthetics and consider the insignificant effect of anterior maxillary teeth on the tongue, they were preserved. It was noted that upon primary ulceration, the patient would aggravate it. Thus, in the initial visits, all irregular surfaces and sharp edges of the teeth were ground to obtain smooth surfaces with no sharp edges (Figures 5 and 6). The ulcers healed right after the first grinding session. The child was cooperative, and grinding was performed without the need for local anesthesia. In addition, she showed no reaction indicative of pain during grinding. As a higher number of teeth erupted, it was noticed that the new sharp edges of the newly erupted teeth did not cause new ulcers. Hence, as the child aged, grinding was performed selectively, and only the sharp edges that could irritate and ulcerate the tongue were ground. The interesting point was that most self-mutilation ulcers occurred when the child was alone and had no specific entertainment. 

Grinding of the teeth significantly decreased the frequency of oral ulcers. Also, preservation of the teeth as much as possible helped preserve the alveolar ridge for future appliance therapy. On the other hand, preserving the four maxillary incisors improved the patient's appearance and satisfied the parents. The time intervals between the grinding sessions were gradually increased. At the 6-month follow-up, all teeth and the oral mucosa were sound, and grinding was no longer required. 

**Figure 7:**
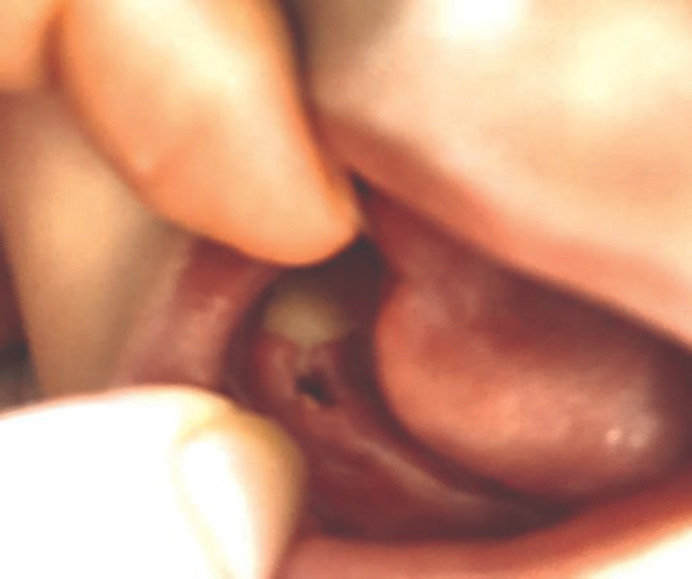
Absence of lower right canine

At the 38-month follow-up, the patient's mandibular right canine tooth was missing. According to her mother, she was playing with the tooth for 2-3 days until she could finally take it out (Figure 7).

## Discussion

HSAN is a rare syndrome with an unknown etiology. Patients often have oral manifestations related to self-mutilation (1,2). At present, diagnosis is currently based on the detection of pathogenic mutations (1). However, in our patient, a primary diagnosis with molecular analysis could not be made because the panel of genes related to neuropathy did not show any pathogenic mutation. However, the SNAP responses were absent in the electrodiagnostic test, which was in favor of the diagnosis of HSAN (3). Oral manifestations often appear following the eruption of primary teeth, involving the lips, the tongue, and buccal mucosa (11,12). Oral manifestations were also present in our patient. Self-mutilation occurs due to the absence of pain sensation. The most common symptom is ulceration of the ventral surface of the tongue due to trauma from mandibular incisors during bottle-feeding. Thus, uncontrollable tongue biting is an important diagnostic symptom (13), which was also the chief complaint of the parents of our patient. 

In some cases, the parents had a third-degree of consanguinity (14). However, further studies are required to extend our knowledge regarding the association of HSANs with consanguine marriage. Variable degrees of mental disability, bone fractures, and chronic bone and joint infections are among other symptoms of HSANs (15). Due to the variations in oral manifestations of HSANs, no standard treatment protocol exists, and the patients are in need of immediate treatment (16). In our patient, only her mandibular anterior teeth and left molar were extracted due to severe trauma to the tongue. Unlike previous reports, selective grinding of other teeth during regular follow-ups was successful, and we preserved both the esthetic facial appearance of the child and her alveolar ridge. In patients with HSAN, the progression of caries and pulpal involvement often occur asymptomatically, with no pain or discomfort. Thus, it is imperative to prevent dental caries by regular follow-ups. Parents play a fundamental role in this respect, and their psychological support is critical to understand the child's condition and to avoid self-mutilation (17,18). They also have a prominent role in the oral hygiene and oral health of their child (14). In the 1960s, the treatment of HSAN was based on the extraction of all primary teeth and the fabrication of a denture. At present, several procedures are practiced, such as grinding of sharp edges and irregular tooth surfaces, composite reconstruction, and use of oral appliances such as a mouthguard. Intraoral appliances cannot be used at a young age. Also, tooth extraction should be the last resort. Such patients should be under regular follow-ups by a dental team during their entire life (4). In our patient, proper follow-ups and preventive treatments, instead of tooth extraction, significantly decreased the frequency of ulcers, improved the appearance of the child, and preserved her alveolar ridge. A noteworthy issue regarding our patient was that she could not be assigned to any of the existing HSAN types according to her signs and symptoms. She may represent a rare variant of HSAN, which needs further investigation.

**In Conclusion, **Patients with HSANs may present a variety of clinical symptoms, among which oral manifestations such as tongue biting can significantly help in early diagnosis. HSANs are rare genetic disorders with no specific treatment. Thus, education of the parents, regular follow-ups, selective grinding, and other supportive treatments are recommended for such patients to preserve their oral and dental health.

## Authors’ contribution

All authors have read and approved the final manuscript. The authors gave their contribution to the drafting and critical review of the article.

## Conflict of interest

The authors declare that they have no conflict of interest.

## References

[1] Swaiman Kenneth, F et al (2018). Swaiman's Pediatric Neurology Principles and Practice.

[2] Gao L, Guo H, Ye N, Bai Y, Liu X, Yu P, Xue Y, Ma S, Wei K, Jin Y, Wen L (2013). Oral and craniofacial manifestations and two novel missense mutations of the NTRK1 gene identified in the patient with congenital insensitivity to pain with anhidrosis. PloS one..

[3] Rozentsveig V, Katz A, Weksler N, Schwartz A, Schilly M, Klein M, Gurman GM (2004). The anaesthetic management of patients with congenital insensitivity to pain with anhidrosis. Paediatr Anaesth.

[4] Elhennawy K, Reda S, Finke C, Graul-Neumann L, Jost-Brinkmann PG, Bartzela T (2017). Oral manifestations, dental management, and a rare homozygous mutation of the PRDM12 gene in a boy with hereditary sensory and autonomic neuropathy type VIII: a case report and review of the literature. Journal of medical case reports..

[5] Eslamian F, Soleimanpour J (1388). Report of two cases of hereditary autonomic neuropathy of type II in one family. Tbzmedmag..

[6] Dyck PJ, Mellinger JF, Reagan TJ, Horowitz SJ, McDonald JW, Litchy WJ (1983). Not 'indifference to pain' but varieties of hereditary sensory and autonomic neuropathy. Brain..

[7] Dyck PJ, Thomas PK, Dick PJ (1993). Neuronal atrophy and degeneration predominantly affecting peripheral sensory and autonomic neurons. Peripheral Neuropathy.

[8] Haga N, Kubota M, Miwa Z (2015). Japanese Research Group on Congenital Insensitivity to Pain Hereditary sensory and autonomic neuropathy types IV and V in Japan. Pediatr Int..

[9] Chen YC, Auer-Grumbach M, Matsukawa S, Zitzelsberger M, Themistocleous AC, Strom TM (2015). Transcriptional regulator PRDM12 is essential for human pain perception. Nat Genet..

[10] Gardner RJM SG (1990). Syndromes affecting the central nervous system.

[11] Axelrod FB, Pearson J (1984). Congenital sensory neuropathies Diagnostic distinction from familial dysautonomia. Am J Dis Child.

[12] Thompson CC, Park RI, Prescott GH (1980). Oral manifestations of the congenital insensitivity-topain syndrome. Oral Surg Oral Med Oral Pathol.

[13] Okuda K, Toshimi A, Miwa T, Hiroki K (2000). Anaesthetic management of children with congenital insensitivity to pain with anhidrosis. Pediatric Anesthesia.

[14] Ofluoglu D, Altin N, Yaman E, İnce EB, Aytepe Z, Tanyeri H (2016). Oral manifestations and prosthetic rehabilitation in hereditary sensory and autonomic neuropathy (HSAN) type IV: a case report. Journal of Istanbul University Faculty of Dentistry..

[15] Okuda K, Arai T, Miwa T, Hiroki K (2000). Anaesthetic management of children with congenital insensitivity to pain with anhidrosis. Paediatr Anaesth.

[16] Limeres J, Feijoo JF, Baluja F, Seoane JM, Diniz M, Diz P (2013). Oral self-injury: an update. Dent Traumatol..

[17] Schalka MM, Correa MS, Ciamponi AL (2006). Congenital insensitivity-to-pain with anhidrosis (CIPA): a case report with 4-year follow-up. Oral Surg Oral Med Oral Pathol Oral Radiol Endod..

[18] Kouvelas N, Terzoglou C (1989). Congenital insensitivity to pain with anhidrosis: case report. Pediatr Dent..

